# The bHLH transcription factor AcB2 regulates anthocyanin biosynthesis in onion (*Allium cepa* L.)

**DOI:** 10.1093/hr/uhac128

**Published:** 2022-06-02

**Authors:** Xiaojie Li, Linjiao Cao, Bangbang Jiao, Haifeng Yang, Changsheng Ma, Yi Liang

**Affiliations:** Beijing Vegetable Research Center, Beijing Academy of Agriculture and Forestry Sciences, Beijing, 100097, China; Key Laboratory of Biology and Genetic Improvement of Horticultural Crops (North China), Ministry of Agriculture, Beijing, 100097, China; Beijing Key Laboratory of Vegetable Germplasm Improvement, Beijing, 100097, China; Beijing Vegetable Research Center, Beijing Academy of Agriculture and Forestry Sciences, Beijing, 100097, China; Beijing Vegetable Research Center, Beijing Academy of Agriculture and Forestry Sciences, Beijing, 100097, China; Vegetable Research Center, Lianyungang Academy of Agriculture Sciences, Yingbin Boulevard, Haizhou District, Lianyungang, Jiangsu, 222000, China; Department of Vegetables, College of Horticulture, Henan Agricultural University, Zhengzhou, Henan, 450046, China; Beijing Vegetable Research Center, Beijing Academy of Agriculture and Forestry Sciences, Beijing, 100097, China; Key Laboratory of Biology and Genetic Improvement of Horticultural Crops (North China), Ministry of Agriculture, Beijing, 100097, China; Beijing Key Laboratory of Vegetable Germplasm Improvement, Beijing, 100097, China

## Abstract

Onion bulb color is a key breeding trait. The red bulb color is caused by the presence of anthocyanins, which are products of the flavonoid synthesis pathway. Research on flavonoid regulation in onion is lagging compared with that in other crops. *AcB2* encodes a basic helix–loop–helix (bHLH) transcription factor, and its transcription is positively associated with anthocyanin accumulation and correlated with the expression of *AcMYB1*, which is an activator in the flavonoid biosynthetic pathway in onion. Phylogenetic analysis showed that AcB2 was grouped into the TRANSPARENT TESTA 8 (TT8) clade of the bHLH IIIf subgroup. The AcB2 protein contained an MYB-interacting region and physically interacted with AcMYB1 in yeast and tobacco leaves. AcMYB1 directly bound to the promoters of *anthocyanidin synthase* (*AcANS*) and *flavonoid 3-hydroxylase 1* (*AcF3H1*) and activated their expression. The coexpression of *AcB2* with *AcMYB1* in *Arabidopsis thaliana* protoplasts dramatically increased the expression of *AcANS* and *AcF3H1* compared with that under the expression of *AcMYB1* alone. Transient co-overexpression of *AcB2* with *AcMYB1* induced anthocyanin accumulation in the epithelial cells of onion bulbs. Complementation of the *Arabidopsis tt8-1* mutant with *AcB2* restored pigmentation defects in *tt8-1*. In addition, AcB2 physically interacted with AtTT2 in yeast cells and tobacco leaves, indicating that the functions of AcB2 were similar to those of AtTT8. Together, these results demonstrated that *AcB2* enhanced the function of *AcMYB1* in upregulating anthocyanin biosynthesis in onion, which provides a theoretical basis for breeding onions with higher anthocyanin contents.

## Introduction

Flavonols, anthocyanins, and proanthocyanidins (PAs) are all metabolites of the flavonoid biosynthetic pathway and accumulate in the seed coats [1–3], flowers [4–6], fruits [7–9], and leaves [10, 11] of many plants. The flavonoid biosynthetic pathway has been well characterized, and the major biosynthetic steps and the corresponding structural genes have been identified in several model crop plants. Onion bulbs range in color from white and pink to red, and these colors result from many different variations and contents of flavonoid compounds [12, 13]. A comparative transcriptional analysis in onion showed that the expression levels of flavonoid structural genes were positively associated with anthocyanin accumulation [14].

Regulation of the flavonoid biosynthetic pathway commonly occurs at the transcriptional level by the MYB-bHLH-WD40 transcription factor (TF) complex [15–17]. The basic helix–loop–helix (bHLH) protein family is the second-largest class of plant TFs and is divided into 26 subgroups [18]. Flavonoid-related bHLHs belong to subgroup IIIf, which contain an MYB-interacting region (MIR) at the amino terminus, a WD40 repeat domain (WDR)-interacting region, and a conserved bHLH domain [17]. The MIR domain interacts with a conserved motif [(D/E)LX2(R-K)X3LX6LX3R] in the R3 repeat of MYBs [19]. Flavonoid-related MYBs usually bind to the promoters of target structural genes to activate their expression, and this binding process depends on the interaction with bHLHs. MBW complexes, such as transparent testa2 (TT2)-TT8-transparent testa glabra1 (TTG1) in *Arabidopsis* [15, 20–22], anthocyanin2 AN2-JAF13-AN11 in *Petunia hybrida* [4, 23], and legume anthocyanin production1 (MtLAP1)-MtTT8-MtWD40-1 in *Medicago truncatula* [24, 25], are necessary for anthocyanin and PA biosynthesis in plants. In *Arabidopsis*, the MBW complex TT2-TT8-TTG1 regulates flavonoid biosynthesis [15], and the *tt8-1* mutant is deficient in flavonoid and PA biosynthesis, resulting in a yellow seed coat [21].

In contrast to those in model plants, studies on the gene regulatory network of anthocyanin biosynthesis in onion are very limited. A previous study showed that *AcMYB1* activated anthocyanin synthesis [26]. The transcript abundance of *AcMYB1* correlated with anthocyanin production. Both transient and stable heterologous coexpression of *AcMYB1* and the anthocyanin bHLH regulator *Zm-Lc* in onion and garlic induced the accumulation of anthocyanins [26]. These results suggested that AcMYB1 controlled anthocyanin accumulation together with its regulatory partner bHLH. However, the bHLH partners of AcMYB1 in onion have not been reported. The identification of onion anthocyanin regulatory factors will provide a basis for the breeding of highly nutritious and high-anthocyanin onions.

In addition, at least five major loci (*I*, *C*, *G*, *R*, and *L*) controlling bulb color in onion have been identified by inheritance research [27–30]. The *I* and *C* loci were found to determine the white bulb color [30, 31]. Recently, the bHLH transcription factor *AcB2* was verified as a causative gene for the *C* locus [32]. A non-autonomous DNA transposon, *AcWHITE*, was found in the promoter of *AcB2* in white onion and linked perfectly to the *C* locus. However, not all white onion lines harbor the *AcWHITE* transposon and, more importantly, there are still knowledge gaps regarding the function of AcB2 in regulating anthocyanin biosynthesis. Clarifying the molecular function of AcB2 is necessary to further reveal the regulatory pathway of onion anthocyanin synthesis and to breed high-anthocyanin onions.

This study reports the functional characterization of *AcB2*. The expression level of *AcB2* was drastically reduced in white Ringmaster bulbs compared with red Xiu-Qiu bulbs. However, the *AcWHITE* insertion was not identified in the *AcB2* promoter of Ringmaster onion. Significant transcription reduction of *AcB2* was found in the inner non-red layers of Xiu-Qiu onion in this study, suggesting that the transcription pattern of *AcB2* was independent of the genotype but correlated with the accumulation of anthocyanins. The bHLH TF AcB2 was grouped with AtTT8 and was located in the nucleus and cytoplasm of tobacco leaf cells. AcB2 physically interacted and cooperated with AcMYB1 to activate the anthocyanin structural genes *AcF3H1* and *AcANS* and promoted anthocyanin accumulation. AcB2 complemented the flavonoid deficiency phenotype of *attt8* and interacted with the *Arabidopsis* MYB TF AtTT2. Consistent with these results, *AcB2* had a function similar to that of *AtTT8* in controlling the anthocyanin biosynthetic pathway.

## Results

### The expression level of *AcB2* was correlated with anthocyanin biosynthesis

**Figure 1:**
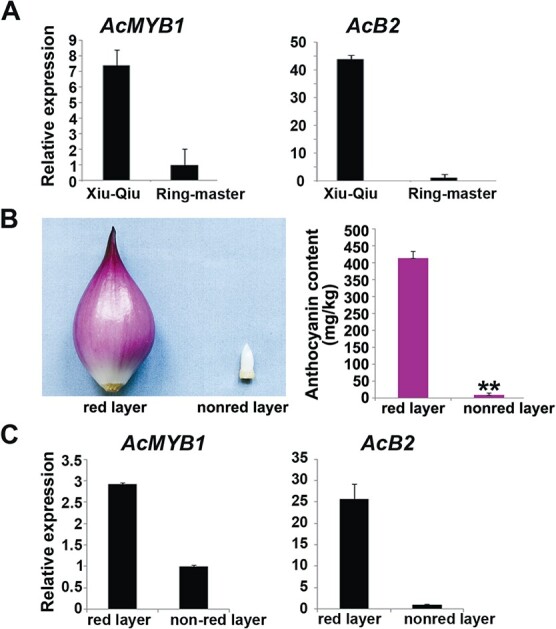
Expression pattern of *AcB2*. qRT–PCR analysis of *AcMYB1* and *AcB2* transcript abundances in (A) Xiu-Qiu red onions and Ringmaster white onions and (C) red and inner non-red layers of Xiu-Qiu red onions. Transcript abundance was normalized to *β-ACTIN* transcript abundance. (B) Pigmentation and anthocyanin content in the red and non-red layers of Xiu-Qiu red onions. All data are from three biological replicates and expressed as mean ± standard deviation. ^**^P < .01 using Student’s *t*-test (*n* = 3).

A comparative transcriptional analysis was performed using Xiu-Qiu (red) and Ringmaster (white) onion bulbs [14]. In addition to the previously identified structural genes, the *AcMYB1* and *AcB2* genes showed drastic expression reduction in the Ringmaster onion bulbs, and the results were verified by quantitative real-time PCR (qRT–PCR; Fig. 1A). Insertion of the DNA transposon *AcWHITE* into the *AcB2* promoter resulted in white-colored onion bulbs. However, the white Ringmaster onion bulbs did not contain an *AcWHITE* insertion, and no polymorphism in this gene was identified between red (Xiu-Qiu) and white (Ringmaster) onion bulbs.

The expression levels of *AcMYB1* and *AcB2* were analyzed in different parts of Xiu-Qiu onion bulbs. The anthocyanin content was reduced dramatically in the inner non-red layers of Xiu-Qiu onion bulbs (Fig. 1B). The qRT–PCR results showed significant transcription reduction of *AcMYB1* and *AcB2* in the inner non-red layers compared with the red layer of Xiu-Qiu onion bulbs (Fig. 1C), indicating that the expression patterns of *AcMYB1* and *AcB2* correlated with anthocyanin biosynthesis.

### *AcB2* encoded a bHLH protein containing an MYB-interacting region

**Figure 2:**
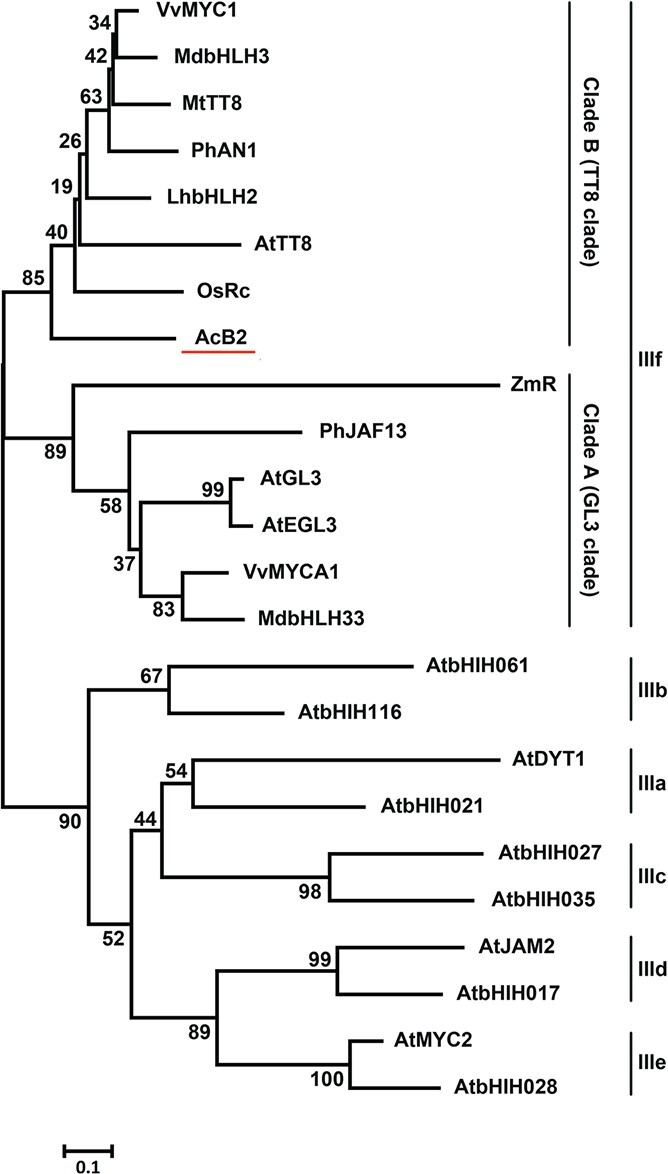
A neighbor-joining phylogenetic tree of bHLH subgroup III TFs. Amino acid sequences were acquired from GenBank with the following accession numbers: AcB2 (AUG71567) in *Allium cepa*; AtTT8 (Q9FT81), AtGL3 (NP_680372), AtEGL3 (Q9CAD0), AtbHLH061 (AAM10950), AtbHLH116 (AAL84972), AtDYT1 (O81900), AtbHLH021 (NP_179283), AtbHLH027 (AAS79544), AtbHLH035 (NP_974948), AtJAM2 (Q9LNJ5), AtbHLH017 (AAM19778), AtMYC2 (Q39204), and AtbHLH028 (AAL55721) in *Arabidopsis thaliana*; MdbHLH33 (ABB84474) and MdbHLH3 (ADL36597) in *Malus domestica*; VvMYCA1 (ABM92332) and VvMYC1 (ACC68685) in *Vitis vinifera*; MtTT8 (KM892777) in *Medicago truncatula*; OsRc (ABB17166) in *Oryza rufipogon*; PhAN1 (AAG25928) and PhJAF13 (AAC39455) in *Petunia hybrida*; LhbHLH2 (BAE20058) in *Lilium* hybrid; and ZmR (P13027) in *Zea mays*. The numbers next to the nodes are bootstrap values from 1000 replications. The tree was constructed to scale, with branch lengths in the same units as the evolutionary distances used to infer the phylogenetic tree (scale bar, 0.1 aa substitution per site).

The 2006-bp full-length cDNA of *AcB2* was isolated and sequenced to verify the open reading frame (ORF). This gene contained a putative ORF of 1848 bp that encoded a peptide of 615 amino acids (aa), including a conserved bHLH domain and an MIR domain. Alignment of the deduced amino acid sequence of AcB2 with bHLH proteins in other plant species (AtTT8 in *Arabidopsis thaliana*, MdbHLH3 in *Malus domestica*, MtTT8 in *M. truncatula*, PhAN1 in *P. hybrida*, and VvMYC1 in *Vitis vinifera*) revealed an N-terminal MIR at 11–194 aa and a conserved bHLH domain at 436–484 aa (Supplementary Data Fig. S1 and Fig. 4A). The conserved MIR domain was a prerequisite for the formation of MBW activation complexes. A phylogenetic tree was constructed using full-length amino acid sequences of AcB2 and 23 other bHLH proteins (Fig. 2). AcB2 belonged to subgroup IIIf clade B (Fig. 2), clustering with AtTT8 (37.52% identity), MtTT8 (47.36% identity), VvMYC1 (46.18% identity), and MdbHLH3 (43.94% identity), which are all direct regulators of anthocyanin genes. Structure and phylogenetic analyses indicated that *AcB2* was a candidate regulatory gene with a function similar to that of *AtTT8* in the anthocyanin biosynthetic pathway.

### Localization of AcB2 in the nucleus and cytoplasm

**Figure 3:**
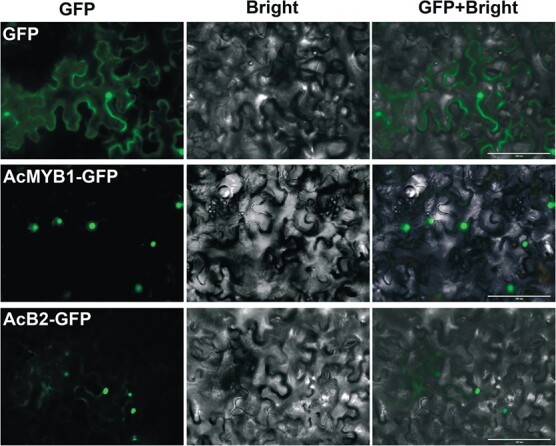
Subcellular localization of AcMYB1 and AcB2 in *N. benthamiana* leaves. The GFP fluorescence signal of the AcMYB1–GFP fusion protein was observed mainly in the nucleus, and the AcB2–GFP fusion protein was detected in the nucleus and cytoplasm. Scale bars = 100 μm.

AcB2 must enter the nucleus of plant cells to function as a TF. A nuclear localization signal peptide (NLS, 444–472 aa) was identified in the predicted AcB2 protein (Supplementary Data Fig. S1) but with a low probability (score = 5.1, using the cNLS Mapper, http://nls-mapper.iab.keio.ac.jp/cgi-bin/NLS_Mapper). A transient expression experiment in *Nicotiana benthamiana* leaves was performed to verify the subcellular localization of AcB2. The green fluorescent signal of AcB2–green fluorescent protein (GFP) was detected using a fluorescence microscope (Fig. 3), showing both cytoplasmic and nuclear localization. In contrast, the GFP control could be found throughout the cells of tobacco leaves. The green fluorescent signal of AcMYB1–GFP was mostly observed in the nucleus, and a weak GFP signal was observed in the cytoplasm. The results indicated that AcB2 and AcMYB1 could be transported to the nucleus.

### The N-terminus of AcB2 physically interacted with AcMYB1

**Figure 4:**
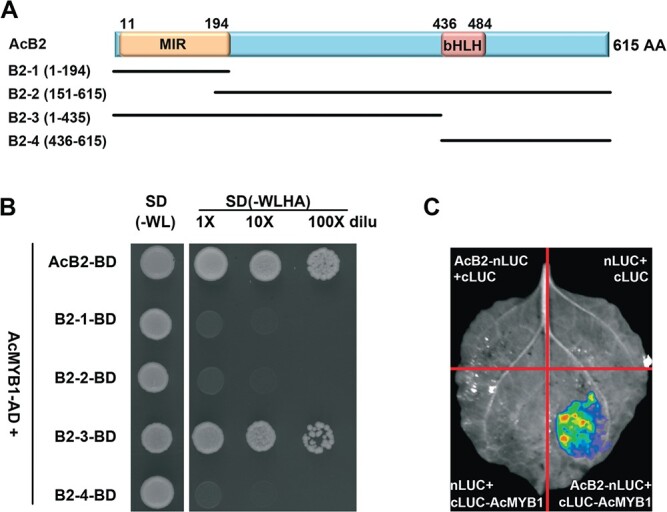
Physical interaction between AcB2 and AcMYB1. (A) Schematic of full-length and truncated AcB2 protein. Coding sequences of AcB2 (1–615 aa), B2-1 (1–194 aa), B2-2 (151–615 aa), B2-3 (1–435 aa), and B2-4 (436–615 aa) were cloned into the BD plasmid, and *AcMYB1* was cloned into the AD plasmid. (B) AcMYB1 physically interacted with AcB2 in the yeast two-hybrid assay. AD, activation domain; BD, binding domain; SD, minimal medium; W, tryptophan; L, leucine; H, histidine; and A, adenine. (C) Luciferase complementation imaging confirming the interaction of AcB2 and AcMYB1.

As putative transcriptional regulators, bHLH proteins interact with MYB TFs to compose the MBW complex. Yeast two-hybrid assays were performed to investigate whether AcB2 could interact with AcMYB1. A full-length AcB2 protein was found to interact with AcMYB1 directly (Fig. 4B). To verify which domain of AcB2 was responsible for interacting with AcMYB1, truncated fragments of B2, B2-1 (1–194 aa), B2-2 (151–615 aa), B2-3 (1–436 aa), and B2-4 (435–615 aa) (Fig. 4A) were used as bait to probe putative interactions with AcMYB1. The results showed that the truncated N-terminus (1–436 aa) of B2 interacted strongly with AcMYB1, while the MIR domain fragment (1–194 aa) did not (Fig. 4B). All these results implied that, in addition to the MIR domain, the middle region of the peptide (194–436 aa) was also required for AcB2 binding to AcMYB1.

Furthermore, a luciferase complementation imaging (LCI) assay was performed in tobacco leaves by transient coexpression of *AcB2* fused with the N-terminus of luciferase (nLUC) and *AcMYB1* fused with the C-terminus of luciferase (cLUC). The results in Figure 4C provide further proof of the interaction between AcB2 and AcMYB1 *in vivo*.

### AcMYB1 bound to *AcAcF3H1* and *AcANS* promoters

**Figure 5:**
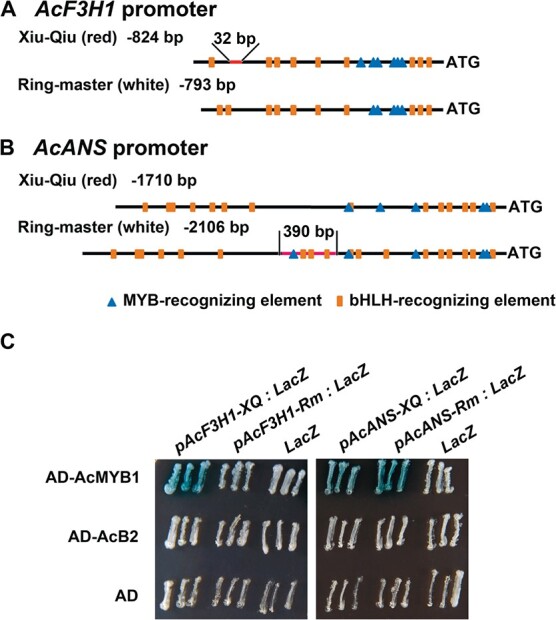
*AcMYB1* bound to the promoters of *AcF3H1* and *AcANS* in yeast cells. (A and B) Pattern diagram of the promoters of *AcF3H1* and *AcANS* from red and white onions. MYB-recognizing elements and bHLH-recognizing elements are shown in the promoters of (A) *AcF3H1* and (B) *AcANS*. Blue triangles, MYB-recognizing elements; orange rectangles, bHLH-recognizing elements; red line, insertion sequence. (C) AcMYB1 bound directly to the *AcF3H1* promoter of red onions and the *AcANS* promoter of red and white onions in yeast one-hybrid assays.

The transcription levels of the structural genes *AcF3H1*, *AcANS*, and *dihydroflavonol 4-reductase-A* (*AcDFR-A*) in the anthocyanin biosynthetic pathway were significantly reduced in white onion compared with red onion [14]. To investigate whether AcMYB1 and AcB2 were involved in the transcriptional regulation of *AcF3H1*, *AcANS*, and *AcDFR-A*, yeast one-hybrid assays were performed. The promoters of these three genes were isolated from Xiu-Qiu and Ringmaster onion bulbs, and the predicted MYB-recognizing element (MRE) ANCNNNC and the bHLH-recognizing element (BRE) CANNTG or CACN(A/C/T)(G/T) [33] were identified (Supplementary Data Figs S2–S4). The number and order of candidate MYB and bHLH sites exhibited marked differences in each gene promoter between Xiu-Qiu and Ringmaster onion bulbs (Fig. 5A and B; Supplementary Data Fig. S5A); therefore, both of them were included in the yeast one-hybrid assays. The results showed that AcMYB1 bound directly to the Xiu-Qiu *AcF3H1* promoter and both the Xiu-Qiu and Ringmaster *AcANS* promoters (Fig. 5C). These results suggested that the 390-bp insertion and the order of different *cis* elements in the Ringmaster *AcANS* promoter did not influence the recognition of AcMYB1.

In comparison, the deletion of a 32-bp region and an MYB site in the *AcF3H1* promoter prevented the recognition of AcMYB1 (Fig. 5C). Sequence analysis of another seven red and four white onion inbred lines showed that the red onion lines R784 and R791 had the same *AcF3H1* sequence as Ringmaster, and the white onion line W559 had the same *AcF3H1* sequence as Xiu-Qiu (Supplementary Data Fig. S6A). These results suggested that the difference in the *AcF3H1* promoter was not linked to onion color and that *AcF3H1* might have been activated by TFs other than AcMYB1. Truncated fragments of the *AcF3H1* promoter (Supplementary Data Fig. S6B) were used in a yeast one-hybrid assay to elucidate which region was responsible for binding to the *AcF3H1* promoter. AcMYB1 failed to bind to any truncated fragments, indicating that the full-length *AcF3H1* promoter contained the major AcMYB1 binding sites.

Yeast one-hybrid assays showed that AcB2 could not bind to the promoter regions of *AcANS* and *AcF3H1* (Fig. 5C). In addition, the promoter of the *AcDFR-A* gene showed self-activation in this yeast one-hybrid system (Supplementary Data Fig. S5B). Thus, more experiments are needed to verify whether AcMYB1 and AcB2 can directly bind to the promoter of *AcDFR-A*.

### AcB2 interacted with AcMYB1 to activate *AcANS* and *AcF3H1*

**Figure 6:**
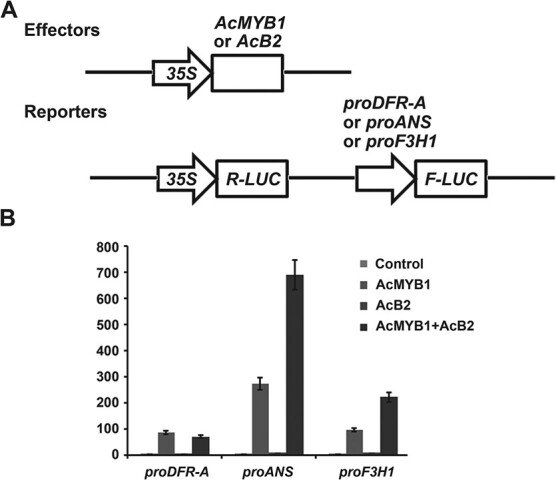
AcB2 interacted with AcMYB1 to activate the *AcANS* and *AcF3H1* promoters*.* (A) Schematic of effector and reporter constructs used in transient expression assays. AcMYB1 and AcB2 driven by the *CaMV 35S* promoter were cloned into the effector construct, and the reporter construct contained the *firefly luciferase* (*F-LUC*) reporter gene driven by the *AcDFR-A*, *AcANS*, or *AcF3H1* promoter and the *Renilla luciferase* (*R-LUC*) gene driven by the *CaMV 35S* promoter as a control for normalization. (B) Promoters of *AcDFR-A*, *AcANS*, and *AcF3H1* were activated by coexpression of *AcB2* and *AcMYB1* in *Arabidopsis* protoplasts. Promoter activities were calculated as the ratio of firefly luciferase activity to *Renilla* luciferase activity. Data are from three independent replicates and expressed as mean ± standard deviation.

Promoter transactivation assays were performed to examine whether the AcMYB1-B2 interaction could activate structural genes. Firefly luciferase reporter constructs were cotransfected with *AcMYB1* or *AcB2* effector constructs into *Arabidopsis* leaf protoplasts (Fig. 6A). *AcANS* was strongly induced by *AcMYB1*, whereas *AcDFR-A* and *AcF3H1* were moderately upregulated by *AcMYB1* (Fig. 6). *AcB2* alone could not activate any of these three structural genes. Coexpression of *AcB2* and *AcMYB1* increased *AcANS* or *AcF3H1* promoter-luciferase reporter activation by >2.5-fold compared with the expression of *AcMYB1* alone. However, *AcB2* could not increase the activation of the *AcDFR-A* promoter induced by *AcMYB1*. These data suggested that AcB2 interacted with AcMYB1 to activate the anthocyanin structural genes *AcANS* and *AcF3H1*.

### Co-overexpression of *AcB2* with *AcMYB1* induced anthocyanin accumulation in onion

**Figure 7:**
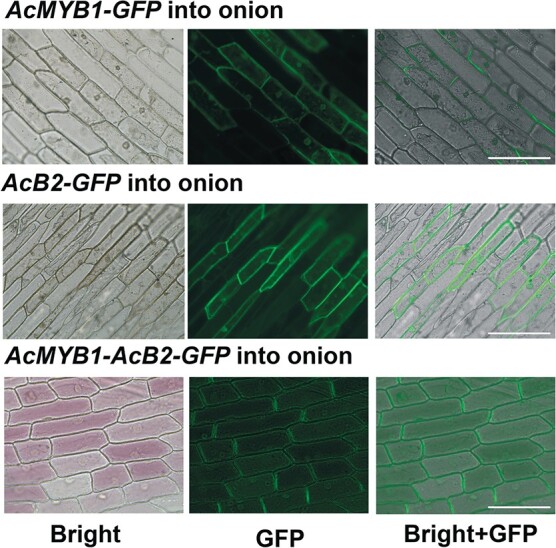
*AcB2* coupled with *AcMYB1* to activate anthocyanin accumulation in onion*. 35S:AcMYB1-GFP*, *35S:AcB2-GFP*, and *35S:AcMYB-AcB2-GFP* were transferred into intraepidermal cells of red onion bulbs using the particle bombardment method. Bright (left), GFP (middle), and merged images were collected. Anthocyanin accumulation was observed with the transient expression of *AcMYB-AcB2*, while *AcMYB1* or *AcB2* alone did not induce a pigment response. Scale bars = 200 μm.

To verify the ability of *AcB2* to upregulate anthocyanin biosynthesis in onion, biolistic transformation was carried out. When *AcMYB1-AcB2-GFP* was transiently introduced into intraepidermal cells of red onion bulbs, anthocyanin accumulation was observed (Fig. 7), while *AcMYB*1 and *AcB2* alone did not activate anthocyanin biosynthesis. In this assay, anthocyanin accumulation was detected within 24 hours after bombardment to prevent autonomous activation of the pigment. The results indicated that AcB2 coupled with AcMYB1 to activate anthocyanin biosynthesis in onion.

### AcB2 interacted with AtTT2 and complemented the *Arabidopsis tt8* mutant phenotype

**Figure 8:**
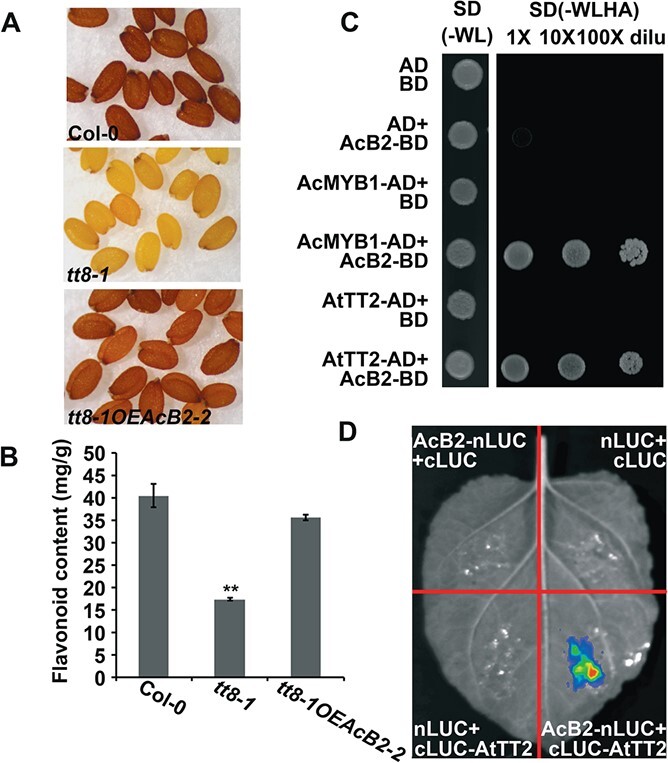
AcB2 complemented the *Arabidopsis* mutant *tt8-1* phenotype and physically interacted with AtTT2. (A) Phenotypic complementation in *tt8-1* mutants expressing *AcB2*. Seeds of wild-type Col-0, *tt8-1*, and *tt8-1OEAcB2-2* (T2 progeny of *tt8-1* homozygotes overexpressing *AcB2*). (B) Flavonoid contents in the seeds of wild-type Col-0, *tt8-1* mutant, and *tt8-1OEAcB2-2*. ^**^P < .01 by Student’s *t*-test (*n* = 3). (C) AcB2 and AtTT2 physically interacted in yeast cells. AD, activation domain; BD, binding domain; SD, minimal medium; W, tryptophan; L, leucine; H, histidine; A, adenine. Images are representative of three independently repeated experiments. (D) Luciferase complementation imaging confirming the interaction of AcB2 and AtTT2.

*AcB2* was overexpressed in the *Arabidopsis tt8-1* mutant (SALK_030966) to test the function of *AcB2* in regulating the biosynthesis of flavonoids. The T2 seed progeny harvested from 11 independent T1 hygromycin B-resistant transgenic lines were isolated, and *AcB2-GFP* expression was detected (Supplementary Data Fig. S7). The *tt8-1OEAcB2-2* transformants had the highest level of expression of *AcB2-GFP* and exhibited brown seeds similar to those of wild-type *Arabidopsis* (Fig. 8A). Furthermore, analysis of flavonoid accumulation in the wild-type and T2 seeds was performed. The data showed that flavonoid deposition in the seeds of the complemented *tt8-1* was similar to that of the wild type (Fig. 8B), suggesting that the flavonoid production defect and yellow seed color of *tt8-1* were restored by AcB2.

Furthermore, yeast two-hybrid assays (Fig. 8C) and LCI assays (Fig. 8D) demonstrated that AcB2 physically interacted with AtTT2. These data demonstrated that *AcB2* had a function similar to that of *Arabidopsis TT8*.

## Discussion

### Regulation of *AcB2* expression by *cis* elements and *trans* factors

Previous studies showed that the transcription patterns of TFs involved in the anthocyanin biosynthetic pathway correlated with anthocyanin accumulation [3, 26]. Baek *et al*. reported that the transcription level of *AcB2* was significantly reduced in white bulbs compared with yellow bulbs [31]. Subsequent research indicated that the insertion of the DNA transposon *AcWHITE* into the *AcB2* promoter resulted in the white color of onion bulbs [32]. *AcWHITE* was not found in the *AcB2* promoter of any yellow or red onion inbred lines. In our study, Ringmaster was a white onion line with a recessive trait [14] that does not possess *AcWHITE* in the *AcB2* promoter. No other polymorphisms were detected in the *AcB2* gene between the Ringmaster and Xiu-Qiu varieties. In addition, the expression of *AcB2* in the inner non-red layer showed a significant reduction compared with that in the red layer of Xiu-Qiu onions, suggesting that the transcription pattern of *AcB2* was independent of the genotype but correlated with the accumulation of anthocyanins and that other genes might regulate the expression of *AcB2*. All the data indicated that the transcription of *AcB2* was regulated by both *cis*-elements and *trans*-factors.

### AcB2 coupled with AcMYB1 to induce the expression of *AcF3H1* and *AcANS*

Previous studies reported that anthocyanin accumulation was spatiotemporally regulated by MYB and bHLH TFs in a variety of plants [34–36]. AcMYB1 is a positive regulator of anthocyanin biosynthesis in onion [26]. *AcB2* was identified as a causal gene for the *C* locus in controlling onion coloration [32]. However, it is still not clear which structural genes are the target genes of AcB2 and AcMYB1. In this study, the transcription patterns of *AcB2* and *AcMYB1* and the structural genes *AcF3H1*, *AcDFR-A*, and *AcANS* were positively correlated with the patterns of anthocyanin biosynthesis in onion. Metabolite profiling and transcriptomic analysis indicated that *AcB2* regulated a subset of flavonoid structural genes.

Yeast one-hybrid assays and promoter transactivation assays showed that *AcF3H1* and *AcANS* were activated by AcMYB1 and enhanced by the presence of AcB2 (Figs 5 and 6). The *cis* elements, such as MREs and BREs, in the promoters of structural genes are essential for regulation by MYBs and bHLHs [33]. The MRE (ANCNNCC) in the *AcF3H1* and *AcANS* promoters had a stronger effect than the MRE (ANCNNAC) in the *AcDFR-A* promoter [33]. The weak transcriptional activation of *AcDFR-A* in the AcMYB1-B2 coexpression system might be due to the weak binding capability of AcMYB1. In addition, previous studies reported that more than one MBW complex regulated anthocyanin biosynthesis in plants. In *Arabidopsis*, both AtTT2-TT8-TTG1 [34] and AtPAP1-TT8-TTG1 [37] complexes transactivated anthocyanin biosynthetic pathway structural genes. The results indicated that other MBW components were involved in *AcDFR-A* transactivation. Significantly, the results showed that AcB2 alone could not bind and activate structural genes or induce anthocyanin biosynthesis (Figs 5–7). Taken together, these findings show that the simultaneous expression and interaction of AcB2 and AcMYB1 were critical for anthocyanin accumulation via transactivated structural genes.

In summary, our results showed that AcMYB1 alone could directly bind the promoters of anthocyanin structural genes and activate the expression of these genes. AcB2 alone could not directly bind the promoters of structural genes and activate their expression. When both AcMYB1 and AcB2 were present, AcB2 promoted the transcriptional activation of structural genes through its interaction with AcMYB1, and the expression of structural genes was increased to higher degrees. For example, in the presence of both AcB2 and AcMYB1, the expression of *AcANS* and *AcF3H1* increased >2.5-fold compared with that under AcMYB1 alone. The increased expression of structural genes ultimately promoted the accumulation of anthocyanins. Our research elucidated the less-studied molecular regulatory mechanism of AcMYB1-AcB2 in anthocyanin accumulation in onion. This study provides a basis for molecular biology research on anthocyanin biosynthesis in onion and provides new opportunities for breeding onions with higher anthocyanin contents.

## Materials and methods

### Plant materials

Two onion cultivars were used in this research: the white onion Ringmaster and the dark-red onion Xiu-Qiu. Hybridization analysis from a previous study showed that Ringmaster had the recessive white onion trait [14]. The onion cultivars were grown in the experimental field of the Beijing Academy of Agriculture and Forestry Vegetable Research Center under natural conditions. Outer layers of mature Xiu-Qiu and Ringmaster bulbs and outer red layers and inner non-red layers of mature Xiu-Qiu bulbs were used in qRT–PCR and anthocyanin content analyses.

The *A. thaliana* Columbia-0 ecotype and the mutant line *tt8-1* (SALK_030966) were obtained from the *Arabidopsis* Biological Resource Center. Homozygote identification for the *tt8-1* mutant was performed by PCR using the primers tt8-1-LP, tt8-1-RP and LBb1.3 (Supplementary Data Table S1). *Arabidopsis* seeds were sterilized in 1.5% (*v*/*v*) sodium hypochlorite for 20 minutes and cold-treated at 4°C for 3 days before sowing. Young seedlings were planted in Murashige and Skoog (MS) medium or in soil in an incubator with a light:dark photoperiod of 16 hours/8 hours, 25–22°C.

### Gene expression analysis and RNA sequencing

Total RNA was isolated from onions using an RNA extraction kit (Tiangen, DP441). Subsequently, cDNAs were reverse-transcribed using PrimeScript IV First-strand cDNA Synthesis Mix (Takara, 6215A). The ORFs of *AcMYB1* and *AcB2* were amplified from cDNAs using the primers AcMYB1-RT-F, AcMYB1-RT-R, AcB2-RT-F, and AcB2-RT-R (Supplementary Data Table S1). qRT–PCR was performed using Power SYBR™ Green PCR Master Mix (Thermo Scientific, CAT# 4368577) on an ABI 7500 instrument. qRT–PCR was performed using *β-ACTIN* [14] as the internal control to determine the expression levels of *AcMYB1* and *AcB2* in the outer layers of mature Ringmaster bulbs and outer red layers and inner non-red layers of mature Xiu-Qiu bulbs. The primers used are listed in Supplementary Data Table S1.

### Measurement of flavonoid and anthocyanin contents

The flavonoid content in the *Arabidopsis* seeds was detected using a plant flavonoid content detection kit (Solarbio BC1330).

The anthocyanin content in onion was detected using a previously described method [38] with slight modifications. Samples (20 mg) were immersed in 600 μL of 1% HCl–ethanol and incubated for 6 hours at 4°C. Subsequently, 200 μL of water and 200 μL of chloroform were added per sample. Following centrifugation at 12 000 × *g* for 10 minutes at room temperature, the supernatant was collected. Optical density (OD) was measured at 530 and 657 nm. The anthocyanin content was calculated using the formula OD530 nm − 0.33 × OD657 nm. Each sample was extracted and measured three times.

### Sequence alignment and phylogenetic analysis

The bHLH-related proteins were selected by BLASTP (https://blast.ncbi.nlm.nih.gov/Blast.cgi) of the NCBI database. Sequence alignment of the protein domain of AcB2 with related bHLH proteins was performed using DNAMAN software. Sequence alignment of the amino acid sequences of proteins used in the phylogenetic analysis was performed using ClustalX2 software. The phylogenetic tree was constructed based on the neighbor-joining method with MEGA software. The node support of the phylogenetic tree was assessed using 1000 bootstrap replicates.

### Yeast two-hybrid assay

Full-length cDNAs of *AcB2*, *AcMYB1*, and *AtTT2* were amplified by reverse transcription RT–PCR using PrimeSTAR Max DNA Polymerase with gene-specific primers. The cDNA of *AcMYB1* and *AtTT2* was introduced into pGADT7 and that of *AcB2* was introduced into pGBKT7. The AcB2-1 (1–194 aa), AcB2-2 (151–615 aa), AcB2-3 (1–435 aa), and AcB2-4 (436–615 aa) truncated fragments were cloned and introduced into pGBKT7. The primers used for plasmid construction are listed in Supplementary Data Table S1. Pairs of constructs with bait and prey plasmids were cotransformed into the AH109 yeast strain. Medium lacking Trp and Leu was used to screen positive strains carrying both pGADT7 and pGBKT7. Independent positive transformants were spotted on medium lacking Trp, Leu, His, and Ade at several dilutions and cultured for 3 days at 30°C for interaction detection.

### Yeast one-hybrid assay

Yeast one-hybrid assays were performed using the method described in a previous study [39]. *AcF3H1* promoters were obtained from Xiu-Qiu and Ringmaster onions using the Genome Walking Kit (Takara 6108). The promoters of *AcANS* and *AcDFR-A* from Xiu-Qiu and Ringmaster onions were amplified using PrimeSTAR Max DNA Polymerase. The primers are listed in Supplementary Data Table S1. The promoters of *AcDFR-A*, *AcANS*, and *AcF3H1* were cloned into pLacZi, and cDNAs of *AcMYB1* and *AcB2* were introduced into pB42AD. The pairs of constructs were cotransformed into the yeast strain EGY48. Positive strains were screened for the development of blue color on a medium lacking Trp and Ura but containing X-gal. The primers used for the construction of pLacZi and pB42AD are listed in Supplementary Data Table S1.

### Luciferase complementation imaging assay

The full-length cDNAs of *AcMYB1* and *AtTT2* were inserted into the plasmid *35S::cLuc* and the full-length cDNA of *AcB2* was inserted into the plasmid 35S::nLuc. The constructs were transferred into *Agrobacterium tumefaciens* strain GV3101. Various pairs of constructs were co-infiltrated into *N. benthamiana* leaves as described in a previous study [40]. Images were acquired using a charge-coupled device camera. The primers used for plasmid construction are listed in Supplementary Data Table S1.

### Subcellular localization of AcB2

Full-length cDNAs of *AcMYB1* and *AcB2* were amplified by reverse transcription RT–PCR and introduced into the modified vector pCAMBIA1300 [41]. The binary construct containing fused *AcMYB1-GFP* and *AcB2-GFP* under the control of a super-promoter was transferred into *A. tumefaciens* strain GV3101. *Agrobacterium*-mediated transformation of *N. benthamiana* leaves was performed as part of an LCI assay. GFP signals were detected 3 days after transformation using a ZEISS LSM710 confocal microscope with excitation at 488 nm and a 522–572 nm filter.

### Transactivation assays

Transfection of *Arabidopsis* protoplasts and promoter transactivation assays were performed as described previously [42]. The promoters of *AcANS*, *AcF3H1*, and *AcDFR-A* were introduced into the reporter plasmid pGreenII 0800 LUC, and cDNAs of *AcMYB1* and *AcB2* were introduced into the effector plasmid pGreenII 62-SK. A *Renilla* luciferase gene in pGreenII 0800 LUC was used as the control. The effector and reporter construct pairs were cotransfected into *Arabidopsis* protoplasts. The luciferase activities were quantified by the Dual-Luciferase Reporter Assay Kit (Vazyme DL101–01). The primers are listed in Supplementary Data Table S1.

### Particle bombardment experiment

*AcMYB1-GFP*, *AcB2-GFP*, and *AcMYB-AcB2-GFP* were cloned into the vector pYBA1132 under the control of the *CaMV 35S* promoter. Particle bombardment using Biolistic PDC-1000/He was carried out as described previously [43]. Intraepidermal skin of red onion bulbs was laid on MS plates for 8 h. The concentration of the constructs in the particles was 0.35 μg/mg gold particles, and 0.6 mg of gold particles was added for each construct. Images of the brightness and GFP signals were detected using an EVOS FL Auto fluorescence microscope 16 hours after bombardment. Each experiment was replicated three times.

### Transformation of *attt8-1*

Full-length cDNA of *AcB2* fused with GFP under the control of a super-promoter was introduced into a modified pCAMBIA1300 vector and transferred into the *A. tumefaciens* strain GV3101. The positive strain was transformed into *attt8-1* using the protocol described in a previous study [43]. The T2 progenies originating from 12 independent T1 hygromycin B–resistant transformants were confirmed by reverse transcription RT–PCR for *AcB2* transcription. The primers are listed in Supplementary Data Table S1.

## Acknowledgements

We thank Junna He (China Agricultural University) for complementation of *Arabidopsi*s *tt8-1*. This research was supported by the Technological Innovation Capacity Program of the Beijing Academy of Agricultural and Forestry Sciences (KJCX20200113), the Innovation and Development Program of the Beijing Vegetable Research Center (KYCX202001-06), and the National Natural Science Foundation of China (No. 32102372).

## Author contributions

X.L. designed the project and planned the experiments. X.L., L.C., and B.J. performed the experiments and analyzed the results. H.Y. and C.M. provided the onion materials. X.L. and Y.L. wrote the manuscript.

## Data availability

The authors confirm that all data that are needed to replicate this study and to draw conclusions are within the paper.

## Conflict of interest

The authors declare no competing interests.

## Supplementary data

Supplementary data is available at *Horticulture Research* online.

## Supplementary Material

Web_Material_uhac128
