# Supplementary material for: The bHLH transcription factor AcB2 regulates anthocyanin biosynthesis in onion (Allium cepa L.)
Source: Hortic Res. 2022 Jun 2;9:uhac128. doi: 10.1093/hr/uhac128 (PMC9418810; doi:10.1093/hr/uhac128)
Supplement: Web_Material_uhac128 [file web_material_uhac128.zip › Supplementary Figures.pdf]

The bHLH transcription factor AcB2 regulates anthocyanin biosynthesis in onion (*Allium cepa* L.)

Xiaojie Li, Linjiao Cao, Bangbang Jiao, Haifeng Yang, Changsheng Ma, Yi Liang

SUPPLEMENTARY FIGURES

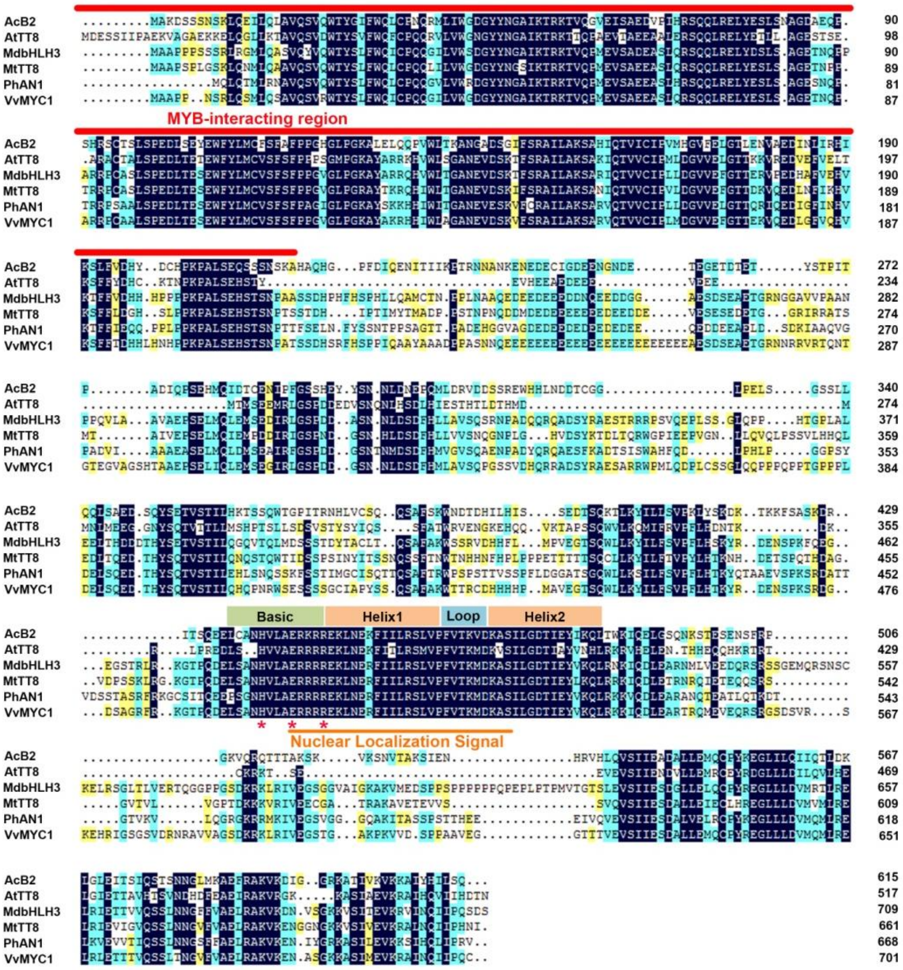

Figure S1. Alignment of amino acid sequences of AcB2 (AUG71567) compared with AtTT8 (Q9FT81), MdbHLH3 (ADL36597), MtTT8 (KM892777), PhAN1 (AAG25928), and VvMYC1 (ACC68685). Red line, MYB-interacting region; box, bHLH domain; orange line, nuclear localization signal; red star, HER motif.

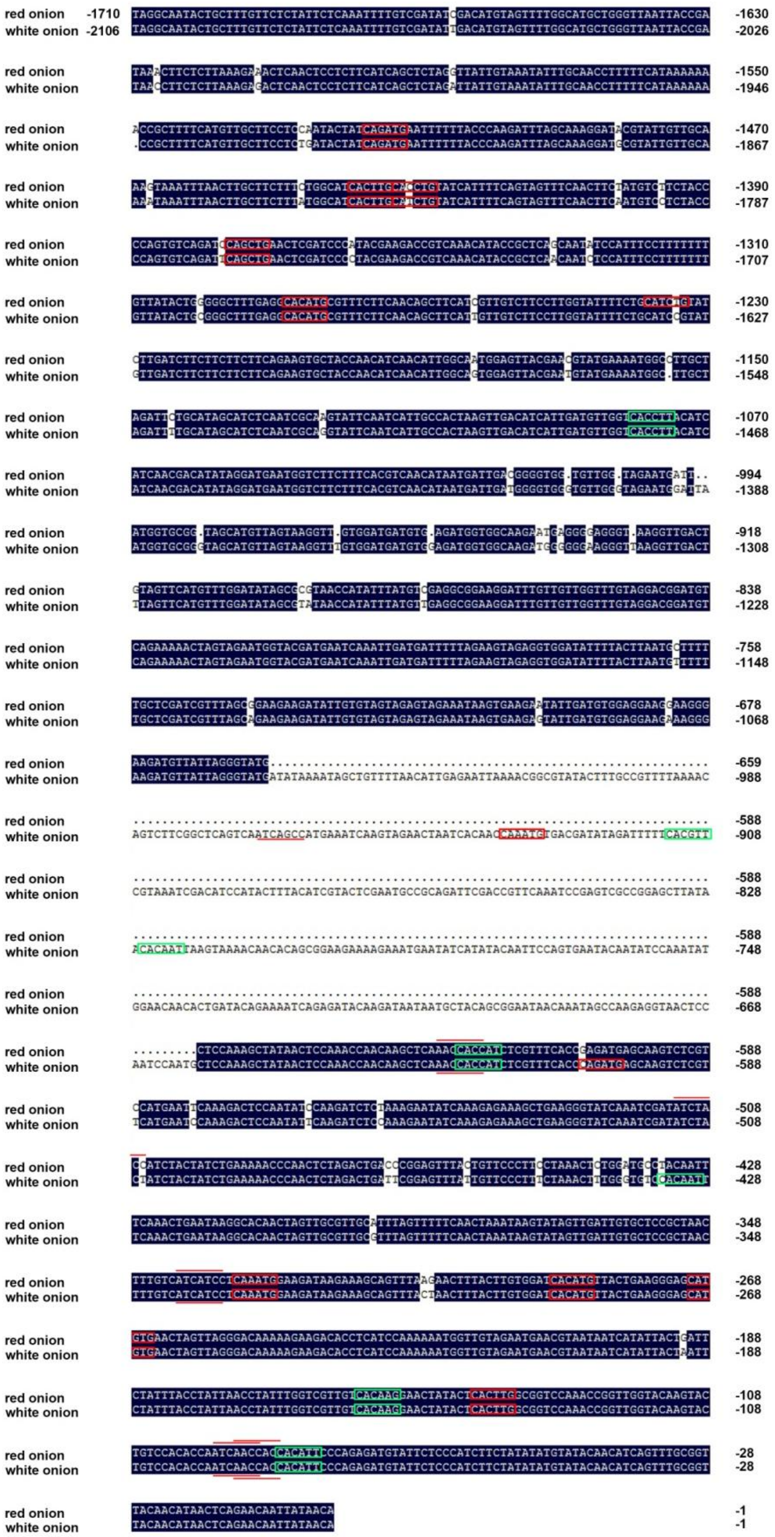

Figure S2. Alignment of *AcANS* promoter nucleotide sequences from red and white onions. Red line, Strong MYB-recognizing element; red box, bHLH-recognizing element CANNTG; green box, bHLH-recognizing element CACN(A/C/T)(G/T); and black dot, nucleotide deletion.

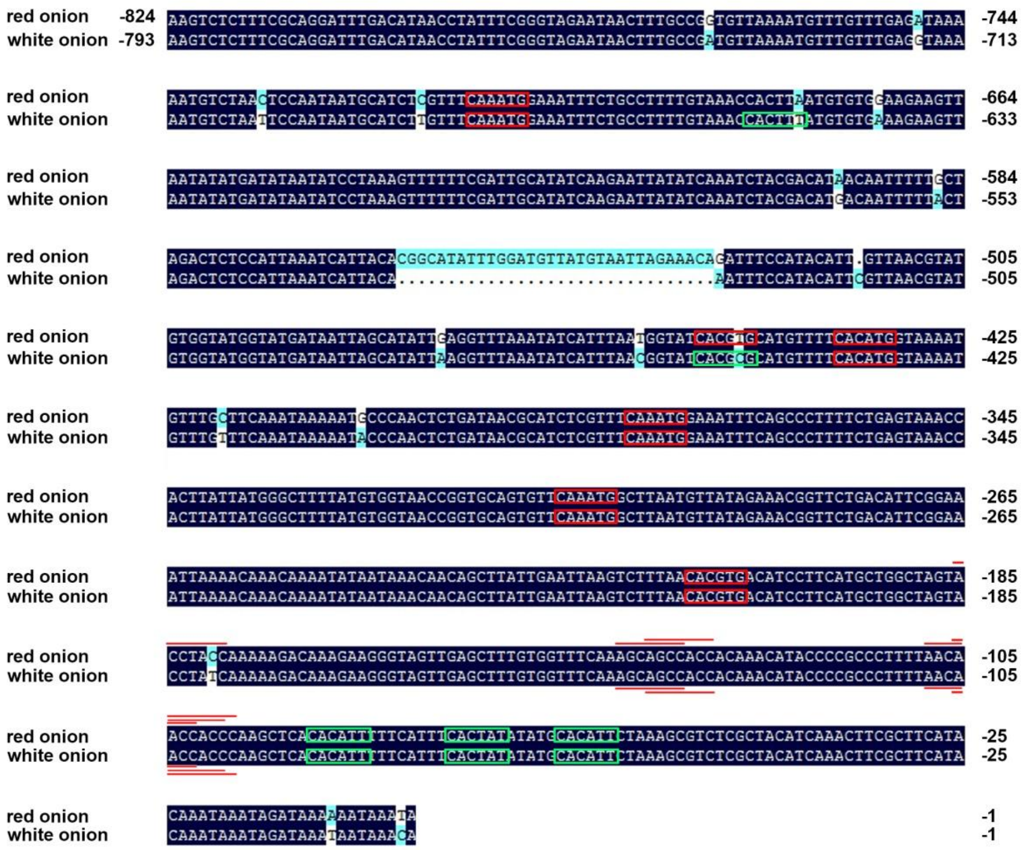

Figure S3. Alignment of *AcF3H1* promoter nucleotide sequences from red and white onions. Red line, Strong MYB-recognizing element; red box, bHLH-recognizing element CANNTG; green box, bHLH-recognizing element CACN(A/C/T)(G/T); and black dot, nucleotide deletion.

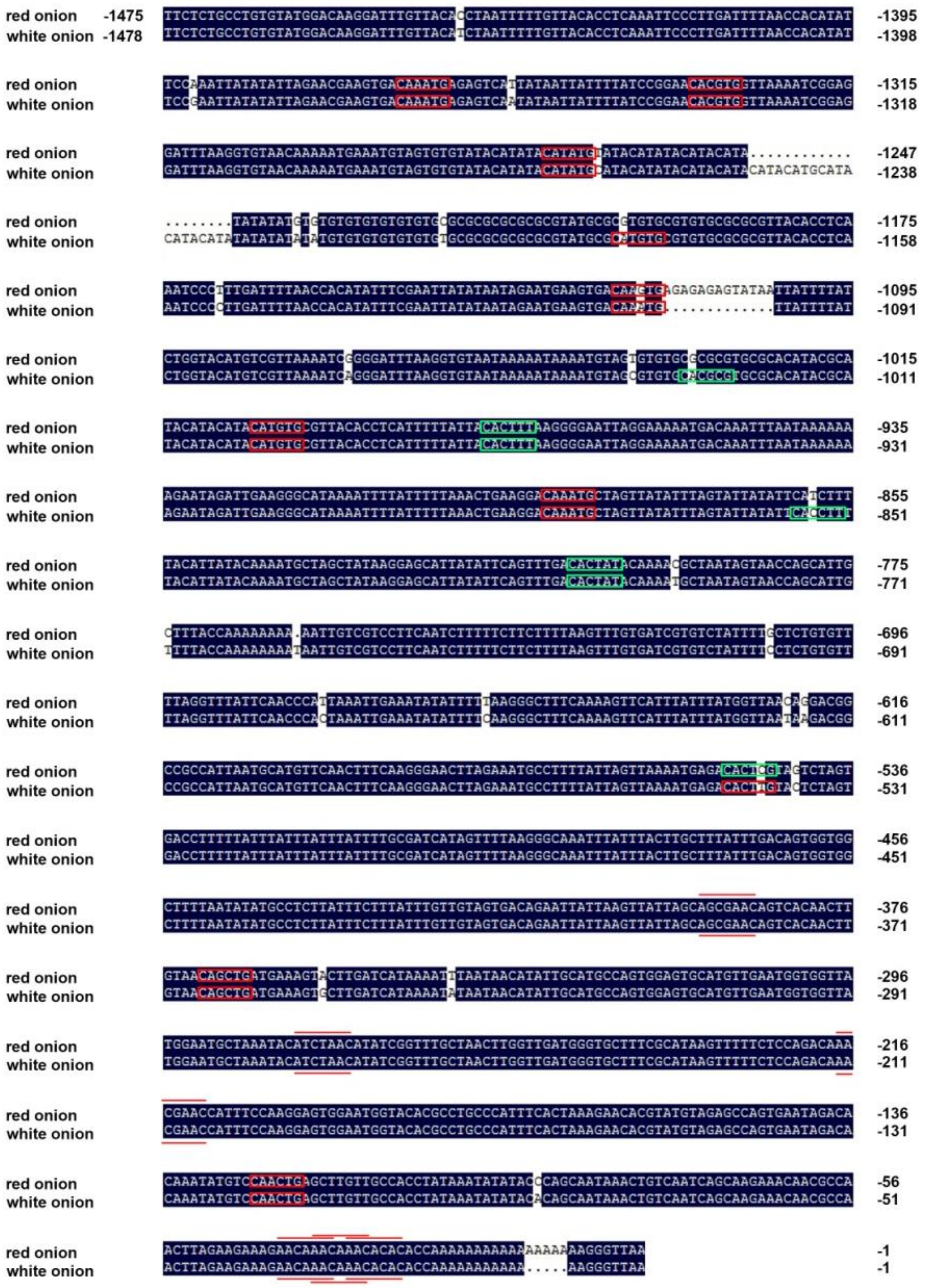

Figure S4. Alignment of *AcDFR* promoter nucleotide sequences from red and white onions. Red line, Weak MYB-recognizing element; red box, bHLH-recognizing element CANNTG; green box, bHLH-recognizing element CACN(A/C/T)(G/T); and black dot, nucleotide deletion.

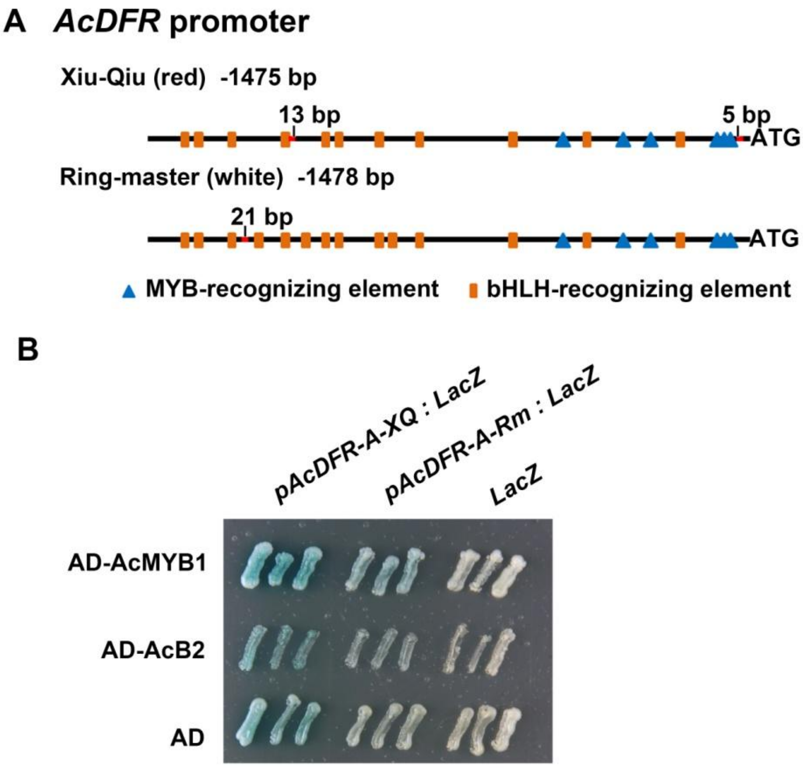

Figure S5. Binding activity analysis of *AcMYB1*, *AcB6*, and the promoter of *AcDFR-A* in yeast cells. (A) MYB-recognizing elements and bHLH-recognizing elements are shown in the promoter of *AcDFR-A*. Blue triangle, MYB-recognizing element; orange rectangle, bHLH-recognizing element; and red line, insertion sequence. (B) Yeast one-hybrid assays of *AcMYB1*, *AcB6*, and the promoter of *AcDFR-A* in red and white onions.

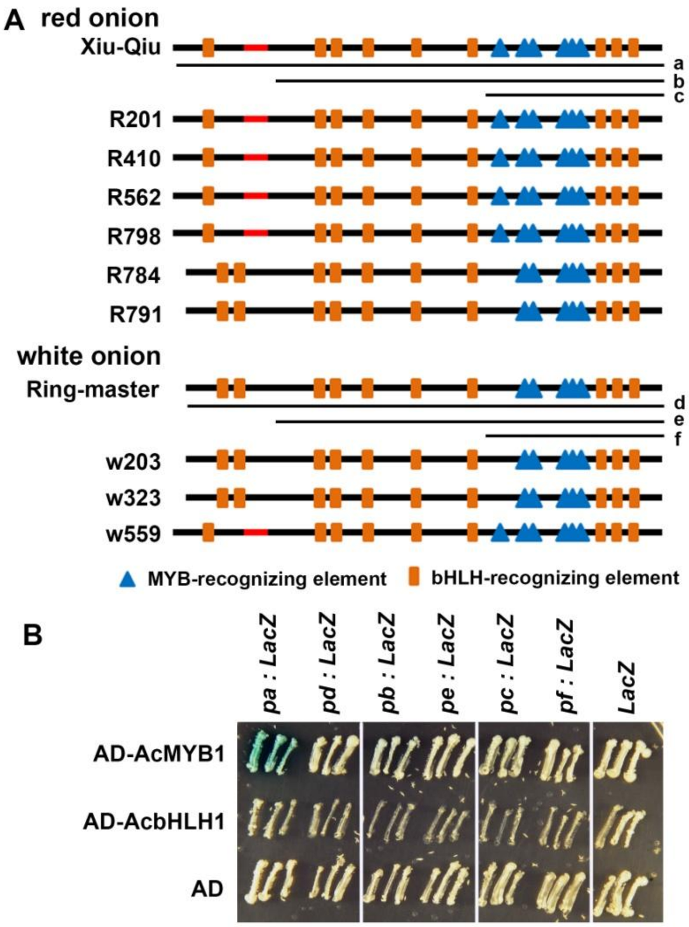

Figure S6. Binding activity analysis of *AcMYB1*, *AcB6*, and the full- and partially truncated promoters of *AcF3H1* in yeast cells. (A) MYB-recognizing elements and bHLH-recognizing elements are shown in the promoter of *AcF3H1* from different inbred lines. Xiu-Qiu R201, R410, R562, R789, R784, and R791 are red onion inbred lines. Ringmaster w203, w323, and w559 are white onions. Sequences of a (1-824 bp), b (1-626 bp), and c (1-216 bp) from Xiu-Qiu and d (1-793 bp), e (1-626 bp), and f (1-216 bp) from Ringmaster were cloned into BD plasmid, and the CDS of AcMYB1 was cloned into the AD plasmid. Blue triangle, MYB-recognizing element; orange rectangle, bHLH-recognizing element; and red line, insertion sequence. (B) Yeast one-hybrid assays of *AcMYB1* and *AcB6*, and the different promoter fragments of *AcF3H1* of red and white onions.

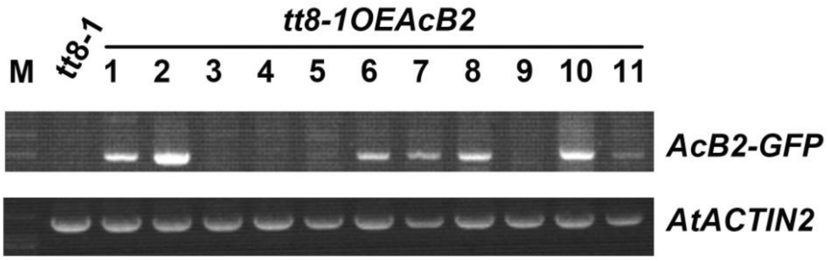

Figure S7. Expression of AcB2-GFP in the T2 seed progeny originating from 11 independent T1 hygromycin B–resistant transformants. *AtACTIN2* was used as an internal control.
