# Supplementary material for: The bHLH transcription factor AcB2 regulates anthocyanin biosynthesis in onion (Allium cepa L.)
Source: Hortic Res. 2022 Jun 2;9:uhac128. doi: 10.1093/hr/uhac128 (PMC9418810; doi:10.1093/hr/uhac128)
Supplement: Web_Material_uhac128 [file web_material_uhac128.zip › supplementary Table.pdf]

The bHLH transcription factor AcB2 regulates anthocyanin biosynthesis in onion (*Allium cepa* L.)

Xiaojie Li, Linjiao Cao, Bangbang Jiao, Haifeng Yang, Changsheng Ma, Yi Liang

Supplementary Table1

tt8-1 mutants identity	
tt8-1-LP:	GGCCCATAAAACGACAAGAAG
tt8-1-RP:	TACCACGTTTTCGTATCTCCG
LBb1.3	ATTTTGCCGATTTCGGAAC
RT-PCR	
AcMYB1-RT-F	ATGTATCACAGCAATTGCA
AcMYB1-RT-R	TTAACTATTAAGTAATTCC
AcB2-RT-F	CTTTGGATATGGCAAAGGACTC
AcB2-RT-R	CTGTGAGAGAATATGGTAAATTGCTT
AcMYB1-qRT-F	TTCTGGAACACTCACCTGAGC
AcMYB1-qRT-R	CGTAAGCAAGCATTCATTTG
AcB2-qRT-F	TATCTGCTGGCGTTATGGGT
AcB2-qRT-R	CAACCCAGTCATAGGTCGTG
β-ACTIN-F	AGAGCAGTATTCCCAAGCATT
β-ACTIN -R	ACACGGCCTGGATAGCAACAT
AtACTIN2-F	AGCACTTGCACCAAGCAGCATG
AtACTIN2-R	ACGATTCCTGGACCTGCCTCATC
Primers in construction of vectors	
MYB1-Sma1-F	CCCCCCGGGATGTATCACAGCAATTGCA
MYB1-BamH1-R	CGCGGATCCTTAACTATTAAGTAATTCC
B2-Ecor1-1-F	CCGGAATTCATGGCAAAGGACTCGAGTAGT
B2-SmaI-1-R	CCCCCCGGGTTACTGTGAGAGAATATGGTA
B2-194-SmaI-R	CCCCCCGGGTTAGAAAAGGCTCTTTATATGTC
B2-151-Ecor1-F:	CCGGAATTCGCCATTCTTGCAAAGAGCG
B2-435-SmaI-R	CCCCCCGGGTTATTCCTCTTGAGAAGTAATTC
B2-436-Ecor1-F:	CCGGAATTCCTATGTGCTAACCATGTAC
TT2-MunI-F	CCGCAATTGATGGGAAAGAGAGCAACTAC
TT2-SalI-R	CGCGTCGACTCAACAAGTGAAGTCTCGGA
F3H-Pro-KpnI-F:	GACCGGTACCAAGTCTCTTTCGCAGGATTTGA
F3H-RED-Pro-SalI-R:	CCGGTCGACTATTTATTTTTTATCTATTTATTTG
F3H-WHITE-Pro-SalI-R:	CCGGTCGACTGTTTATTATTTATCTATTTATTTG
F3H-Pro-626-KpnI-F:	GACCGGTACCATCAAGAATTATATCAAATCTACG
F3H-Pro-216-KpnI-F:	GACCGGTACCCACGTGACATCCTTCATGCTG
ANS-Pro-KpnI-F:	GACCGGTACCTAGGCAATACTGCTTTGTTCTC
ANS-Pro-SalI-R:	CCGGTCGACTGTTATAATTGTTCTGAGTTATG
DFR-Pro-KpnI-F:	GACCGGTACCTTCTCTGCCTGTGTATGGACA
DFR-Pro-SalI-R:	CCGGTCGACTTAACCCTTTTTTTTTTTTT

MYB1-BbsEcoI-F:	CTCGAAGACAGAATTCATGTATCACAGCAATTGCA
MYB1-BbsXhoI-R:	CTCGAAGACACTCGAGTTAACTATTAAGTAATTCC
B2-SalI-R:	CCGGTCGACTTACTGTGAGAGAATATGGTA
MYB1-HindIII-F:	CGCAAGCTTATGTATCACAGCAATTGCA
MYB1-KpnI-TAA-R:	CGGGGTACCACTATTAAGTAATTCCTCC
B2-BsaIHindIII-F:	CGCGGTCTCAAGCTTATGGCAAAGGACTCGAGTAGT
B2-KpnI-TAA-R:	CGGGGTACCCTGTGAGAGAATATGGTAAAT
